# Incidence and Factors Associated with Postspinal Headache in Obstetric Mothers Who Underwent Spinal Anesthesia from a Tertiary Hospital in Western Uganda: A Prospective Cohort Study

**DOI:** 10.1155/2023/5522444

**Published:** 2023-08-10

**Authors:** Mohamud Jelle Osman, Joy Muhumuza, Yarine Fajardo, Andrew Kwikiriza, Baluku Asanairi, Rogers Kajabwangu, Marie Pascaline Sabine Ishimwe, Theoneste Hakizimana

**Affiliations:** ^1^Department of Obstetrics and Gynecology, Faculty of Clinical Medicine and Dentistry, Kampala International University, Western Campus, Ishaka, Uganda; ^2^Department of Anesthesia, Faculty of Clinical Medicine and Dentistry, Kampala International University, Western Campus, Ishaka, Uganda; ^3^Faculty of General Medicine, University of Rwanda, Kigali, Rwanda

## Abstract

**Background:**

The proportion of obstetric mothers reporting postspinal headache (PSH) in Uganda is high. The aim of this study is to determine the incidence and factors associated with postspinal headache among obstetric patients who underwent spinal anesthesia during cesarean section at a tertiary hospital in Western Uganda.

**Methods:**

A prospective cohort study was done on 274 consecutively enrolled obstetric patients at Fort Portal Regional Referral Hospital (FRRH) from August to November 2022. Pretested questionnaires were used to obtain the data needed for analysis. The data were entered into Microsoft Excel version 16, coded, and transported into SPSS version 22 for analysis. Descriptive statistics was used to determine the incidence of postspinal headache. Binary logistic regression was computed to obtain factors associated with postspinal headache.

**Results:**

The overall incidence of postspinal headache was 38.3% (95% CI: 32.5–44.4). Factors with higher odds of developing postspinal headache included using cutting needle (^a^OR 3.206, 95% CI: 1.408–7.299, *p*=0.006), having a previous history of chronic headache (aOR 3.326, 95% CI: 1.409–7.85, *p*=0.006), having lost >1500 mls of blood intraoperatively (^a^OR 6.618, 95% CI: 1.582–27.687, *p*=0.010), initiation of ambulation >24 h after spinal anesthesia (^a^OR 2.346, 95% CI: 1.079–5.102, *p*=0.032), allowing 2-3 drops of cerebrospinal fluid (CSF) to fall (aOR 3.278, 95% CI: 1.263–8.510, *p*=0.015), undergoing 2 puncture attempts (^a^OR 7.765, 95% CI: 3.48–17.326, *p* ≤ 0.001), 3 puncture attempts (^a^OR 27.61, 95% CI: 7.671–99.377, *p* ≤ 0.001) and >3 puncture attempts (^a^OR 20.17, 95% CI: 1.614–155.635, *p*=0.004), those prescribed weak opioids (^a^OR 20.745, 95% CI: 2.964–145.212, *p*=0.002), nonsteroidal anti-inflammatory drug (NSAID) with nonopioids (^a^OR 6.104, 95% CI: 1.257–29.651, *p*=0.025), and NSAID with weak opioids (^a^OR 5.149, 95% CI: 1.047–25.326, *p*=0.044). Women with a body mass index (BMI) of 25–29.9 kg/m^2^ (^a^OR 0.471, 95% CI: 0.224–0.989, *p*=0.047) and a level of puncture entry at L3-4 (^a^OR 0.381, 95% CI: 0.167–0.868, *p*=0.022) had lower odds of developing PSH.

**Conclusions:**

The incidence of postspinal headache is still high as compared to the global range. This was significantly associated with needle design, amount of cerebro-spinal fluid lost, number of puncture attempts, body mass index, previous diagnosis with chronic headache, amount of intraoperative blood loss, time at start of ambulation, level of puncture entry, and class of analgesic prescribed. We recommend the use of a smaller gauge needle, preventing CSF loss, deliberate attempts to ensure successful puncture with fewer attempts, puncture attempts at L3-4, reducing intraoperative blood loss, earlier ambulation, and prescribing adequate analgesia to reduce the incidence of postspinal headache.

## 1. Introduction

Spinal anesthesia was introduced in 1898 by Dr. August Bier and has since become the global anesthesia of choice for operations below the umbilicus as it is cost-effective, safe, and reliable [1], but it is associated with various debilitating complications including postspinal headache [2]. The global incidence ranges between 5–30% [3], but some countries still report higher incidences. In Ethiopia, one study reported an incidence of 42.6% [4] whereas in Uganda, an incidence of 48.8% was reported [5].

As early as 1956, the use of small-gauge needles showed a significant reduction in postspinal headache yet, recent studies still report high incidences [6]. The high incidences of postspinal headache are associated with young age [7], female gender, pregnancy, cutting spinal needle design [8], prior history of postspinal headache, needle orientation perpendicular to dural fibers [9], bigger size of the spinal needle [10], low patient's body mass index, greater number of puncture attempts, sitting position during anesthesia [11], greater amount of cerebrospinal fluid drops allowed to fall [12], prior history of spinal anesthesia, prior history of chronic headache [13], late time at the start of postoperative ambulation [5], level of puncture entry above L3-4 [13] and a loss of more than 500 ml of blood during surgery [5] among others, majority of which are modifiable. Postspinal headache is associated with significant morbidity and mortality and other socioeconomic consequences such as litigation, longer hospital stay, financial burden, reduced productivity, and interruption of maternal and neonatal bonding [13, 14]. Uganda is among countries reporting high incidences of postspinal headache [5]. Unpublished data from the theatre register and a survey done at Fortportal Regional Referral Hospital postnatal ward between March 2022 and June 2022 showed that more than 90% of obstetric operations are done under spinal anesthesia using 25 G cutting spinal needle and about 50% develop postspinal headache. Regardless of the high number of operations done under spinal anesthesia alittle is known about postspinal headache. On this note, there was a need for a study that assessed the incidence and factors associated with postspinal headache in obstetric patients at FRRH.

## 2. Materials and Methods

### 2.1. Study Design and Setting

This was a prospective cohort study that consecutively enrolled obstetric mothers in their immediate postpartum period (within 12 hours after undergoing successful spinal anesthesia during a cesarean section). The study was conducted from postnatal ward, Department of Obstetrics and Gynecology of Fort-Portal Regional Referral Hospital from August 2022 to November 2022. The study participants came from catchment areas of Fort-Portal Regional Referral Hospital such as Kabarole, Bundibugyo, Bunyangabu, Kyegegwa, Kamwenge, Kasese, Ntoroko, and Kyenjojo districts and to a minor extent from other districts of Uganda, DR Congo, Rwanda, and Tanzania. Fort Portal Regional Referral Hospital is a public hospital funded by the Uganda Ministry of Health with a bed capacity of about 350 beds distributed between the different departments including obstetrics and gynecology, internal medicine, surgical, oncology unit, psychiatric, pediatrics, accident and emergency, laboratory, mortuary, hematology, and the imaging department. It serves as a teaching satellite center for Kampala International University (KIU) and internship center for the Ugandan Medical Fraternity and offers 24/7 outpatient and inpatient services in the different departments.

The Department of Obstetrics and Gynecology of FRRH has 105 beds and records around 480 deliveries in a month translating to 10–20 deliveries a day and about 220 operations in 1 month, the majority being obstetric patients. The department is manned by highly skilled staff with a passion for maternal and child health coming both from KIU-Western Campus and the Ministry of Health including 2 professors, 9 consultant obstetricians and gynecologists, 6 anesthetists, 5 senior house officers, 2 special grade medical officers, 3 medical officer, 5 interns, 13 midwives, and 12 nurses among other staff. The study participants were consecutively enrolled until we reached the desired sample size, and follow-up was done for 7 days.

### 2.2. Data Collection

The data were collected by trained midwives together with the principal investigator from patient charts and recordings using a pretested questionnaire. The data collection tool was pretested at the Kampala international University Teaching Hospital which provides obstetric and gynecological services. The adjustments needed were done in the data collection instruments. Patients were interviewed daily regarding the development of any headache and associated symptoms such as neck stiffness, nausea, vomiting, photophobia, tinnitus, and partial hearing loss. Those in the wards were seen at least once daily and interviewed for the development of postspinal headache and its clinical presentation. Those who were discharged before day 7 postspinal were called once daily for the same. The patient who reported bilateral, frontal-occipital headache within those 7 days which worsened on patient assuming upright position or Valsalva but improved on lying flat was suspected to have a postspinal headache.

### 2.3. Sampling Size Determination

The sample size was calculated using by Kish Leslie formula (1999).

(1) $$\begin{array}{c} n=\frac{Z_{\left(\alpha /2\right)}^{2}*\;p\left(1-p\right)}{d^{2}}\text{,} \end{array}$$

where *n* = sample size estimate of study participants, *Z*_(*α*/2)_ = the abscissa of the normal curve that cuts off an area at the tails (standard *Z* value at 95% confidence interval),  = 1.96, *p* = is the estimated proportion of an attribute that was present in the population (incidence of women with PSH = 20.35% at Aga Khan University Hospital, in Nairobi, Kenya [14], and *d* = is the desired level of precision; 5%

(2) $$\begin{array}{c} n=\frac{1.96^{2}*\;0.2035\left(1-0.2035\right)}{0.05^{2}}\\ =249\text{.} \end{array}$$

By adding 10% loss of follow-up/nonrespondents, we got 274 participants.

### 2.4. Inclusion and Exclusion Criteria

We included all obstetric patients who underwent successful spinal anesthesia during cesarean section. All women with life-threatening postoperative complications, unconscious patients, and those who did not have working telephone numbers were not included in the study.

### 2.5. Diagnosis of Postspinal Headache

The clinical criteria for diagnosis of postspinal headache were used as that complication of spinal anesthesia in which a patient developed a bilateral frontal or occipital pain that was throbbing, dull in nature, of varying in intensity, typified by the pain getting worse when the patient sat up and/or stood and/or followed by Valsalva maneuvers such as coughing, straining, and sneezing and improved on lying down within 7 days after spinal anesthesia. Numerical Rating Scale was used while interviewing mothers where the score of 1 to 3 was denoted as mild headache, 4 to 7 as moderate headache, and finally 8 to 10 as severe headache.

### 2.6. Data Processing and Analysis

The data on the checklist were entered in Microsoft Excel version 16, coded, and thereafter imported into SPSS version 22 for analysis. The incidence of postspinal headache was calculated as the number of mothers who developed PSH out of all mothers recruited in the study. This was expressed as frequency and percentages and presented using a pie chart. The factors associated with postspinal headache in obstetric patients who underwent spinal anesthesia at FRRH maternity were determined using both bivariate and multivariate logistic regression analyses. At the bivariate level, factors that had a *p* value ≤0.2 were taken to multivariate analysis. After multivariate analysis, variables with a *p* value ≤0.05 were considered statistically significant.

## 3. Results

### 3.1. Incidence of Postspinal Headache in Obstetric Mothers Who Underwent Spinal Anesthesia at a Tertiary Hospital in Western Uganda

**Figure 1:**
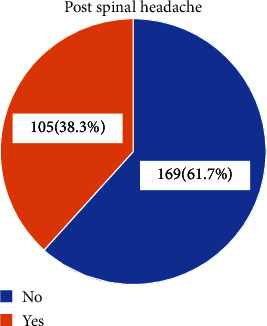
Incidence of postspinal headache in obstetric patients who underwent spinal anesthesia.

A total of 274 mothers in the immediate postnatal ward were recruited for the study with a response rate of 100%. The overall incidence of postspinal headache was 38.3% (95% CI: 32.5–44.4). 61.7% did not develop a postspinal headache (Figure 1).

### 3.2. Factors Associated with Postspinal Headache in Obstetric Mothers Who Underwent Spinal Anesthesia from a Tertiary Hospital in Western Uganda

This study revealed that body mass index (BMI), history of chronic headache, amount of blood loss, start of ambulation, class of analgesia prescribed, number of attempts, CSF allowed to drop, the level of entry, and the design of needle were independent factors associated with postspinal headache. Precisely, women whose BMI ranged from 25 to 29.9 were 0.5 times less likely to develop PSH (^a^OR 0.471, 95% CI: 0.224–0.989, p 0.047) while those who reported a history of chronic headache were 3 times more likely to develop PSH (^a^OR 3.326, 95% CI: 1.409–7.850, *p*=0.006). During operation, women who lost more than 1500 ml of blood were 7 times more likely to develop PSH (^a^OR 6.618, 95% CI: 1.582–27.687, *p*=0.010), and those who started ambulation >24 hours postoperatively were 2 times more likely to develop PSH (^a^OR 2.346, 95% CI: 1.079–5.102, *p*=0.032). Women who were prescribed with weak opioids (^a^OR 20.745, 95% CI-2.964–145.212, *p*=0.002), NSAIDs/Nonopioids (^a^OR 6.104, 95% CI: 1.257–29.651, *p*=0.025), and NSAIDs and weak opioids (aOR 5.149, 95% CI: 1.047–25.326, *p*=0.044) were, respectively, 21, 6, and 5 times more likely to develop PSH as compared with those who were prescribed with NSAIDs and strong opioids. While giving spinal anesthesia, women who had 2 puncture attempts (^a^OR 7.765, 95% CI: 3.48–17.326, *p* < 0.001), 3 puncture attempts (^a^OR 27.61, 95% CI: 7.671–99.377, *p* < 0.001), and more than 3 puncture attempts were, respectively 7, 28, and 20 times more likely to develop PSH as compared with those who had one attempt, likewise women who lost 2-3 drops of CSF during injection of spinal anesthesia (^a^OR 3.278, 95% CI: 1.263–8.510, *p*=0.015) were 3 times more likely to develop PSH while those whose level of entry of needle was L3-4 (^a^OR 0.381, CI: 0.167–0.868, *p*=0.022) were 0.4 less likely to develop PSH and finally those in whom cutting needle (^a^OR: 3.206, 95% CI: 1.408–7.299, *p*=0.006) was used were 3 times more likely to have developed PSH (Table 1).

## 4. Discussion

The overall incidence of PSH among obstetric patients undergoing spinal anesthesia at Fort Portal Regional Referral Hospital was 105 (38.3%). It was analogous to 38.8% reported by Aregawi et al. [15] and 31.3% reported by Ferede et al. [16] in Ethiopia. Although it is higher than 28.8% obtained from a study done by Weji et al. [12] in Ethiopia, 20.35% reported by Gisore et al. [14] at Aga Khan University Hospital in Kenya, 17.1% from the study done by Fearon et al. [17] in Jamaica, and 14.9% obtained from the study done by Bhusal et al. [18] in Nepal, the difference is attributed to different sample sizes, sampling techniques, study designs, settings, and population characteristics. In contrast to the current study, the study done by Weji et al. used the sample size of 150 participants compared to 274 participants in our study which may explain the slightly lower rate in their case.

The difference with the study done in Kenya at the Aga Khan University Hospital was attributed to the staff and study setting [14]. Aga Khan University Hospital is a private facility in the urban setting with an international reputation in individualized care using consultants and a registrar in anesthesia to undertake the procedure as compared to Fort Portal Regional Referral Hospital, a public hospital in rural setting with 83.6% of anesthesia team being diploma holders. The study in Jamaica by Fearon et al. used a sample size of only 70 participants enrolled using the purposive sampling method which is prone to a selection bias. In addition to that, the research excluded those under 18 years and those with chronic headache [17]. In the Bharatpur Hospital, Nepal study, both males 26 (9.2%) and females 256 (90.8%) of all age groups were included in the study with males known to be a low-risk group [18].

The incidence of PSH in this current study is lower than 48.8% obtained in a study done by Nambooze et al. [5] at Mulago National Referral Hospital Uganda, and 42.6% from the study done by Tarekegn et al. [4] at Felege Hiwot Referral Hospital, Bahir Dar. This discrepancy may be attributed to the differences in preoperative hydration, study settings, and the size of the needle used in majority of cases. In this particular study, it was done in a rural setting as compared to the study done in Mulago, Kampala. In the study done by Tarekegn et al., it was noted that the bigger needle sizes (G20 and G21) were used in the majority of their cases (47.8%) as compared to the G25 needle used in 84.7% of our study participants.

This study found that women who underwent spinal anesthesia using cutting spinal needle were 3 times more likely to develop PSH (*p*=0.006). A similar finding was observed in a meta-analysis done by Xu et al. in China [10] and a prospective cohort study done by Weji et al. in Ethiopia [12]. The explanation for the association is that a cutting (traumatic) needle transects the dural fibers, resulting in the dural fiber recoiling backward, thus creating a larger hole compared with pencil-point (atraumatic) which only separates the dural fibers, whereas in pencil-tip, these fibers easily come back together to close the defect. At the Universal College of Medical Science Nepal, 60 patients were randomized to G27 cutting and G27 pencil type each and followed for the development of PSH. It was found that those assigned to G27 cutting were 33% more likely to develop PSH as compared to the other group. This was however not statistically significant (*p*=0.2345) [19].

This study found that women in whom 2-3 drops of CSF were lost were 3 times more likely to develop PSH as compared to those who lost 0-1 drop of CSF. This is similar to a study done in Ethiopia by Weji et al. [12]. In a study done in Nepal, it was reported that there was no association between CSF leakage (*p*=0.204) and PSH development [18], although the size of the needle used was not mentioned. The difference may be explained by the protective techniques used where 194 (68.8%) of puncture attempts were by single attempt and 238/282 (84.4%) with needle parallel to dural fibers as compared to the current study.

This study found that women who had 2, 3, and >3 puncture attempts were, respectively, 8, 28, and 20 times more likely to develop PSH as compared to those who had 1 attempt. This is consistent with a study done in Cuba [20] and the one done by Tarekegn et al. [4] in Ethiopia. The number of puncture attempts is a reflection of the magnitude of damage that is caused, resulting in more sites from which CSF is lost; therefore, fewer attempts result in a lower incidence of PSH. Our findings are in disagreement with the study done in Jamaica by Fearon et al. who reported no association between the number of puncture attempts and PSH [17]. This may be explained by the fact that the majority of their participants 45 (64.35%) required one attempt in addition to the purposive sampling method used which is judgemental with risk selection bias.

This study found that participant with a body mass index of 25–29.9 kg/m^2^ were 0.5 times less likely to develop PSH (*p*=0.047) as compared to those with BMI of 18.5–24.5 kg/m^2^. This is consistent with a study done in Bharatpur Hospital, Nepal [18]. Our findings are different from a study done by Hashemi et al. in Iran [21]. This difference may be attributed to their study population characteristics. A majority of their study participants were obese 179 (52.2%) with BMI > 30 kg/m^2^ and only 164 (47.8%) had a BMI < 30 kg/m^2^ compared to this study where only 26 (9.5%) had a BMI > 30 kg/m^2^.

This study found that women who had lost >1500 ml of blood were 7 times more likely to develop PSH (*p*=0.01) as compared to those who lost <500 ml. This study is consistent with observations made by Nambooze et al. in a research done at Mulago National Referral Hospital, Uganda [5]. The relationship between blood loss and appearance of PSH may be due to cerebral vasodilation that comes with hypotension during episodes of hypovolemic shock; hence, preferential blood supply to the brain is enhanced (brain spearing effect). The cerebral vasodilatation is the newer mechanism for explaining pathophysiology of PSH in line with Monroe–Kellie doctrine.

This study found that participants with prior chronic headache were 3 times more likely to develop PSH (*p*=0.006), compared to those who reported no history of headache. This is consistent with the study done by Jabbari et al. [13] in his review of articles and the one done by Frias Carrazana et al. in Cuba [20]. The precise reason for this is unknown, but it is speculated that there could be some unique physiologic and neurologic transmitters that could explain the risk.

This study found that women who started postoperative ambulation >24 hours after administration of spinal anesthesia were 2 times more likely to develop PSH (*p*=0.032) as compared to those who started ambulating within 12−24 hours of spinal anesthesia. This is consistent with findings in a study by Nambooze et al. in Uganda [5]. Our findings are in disagreement with the study done at Bharatpur Hospital, Nepal, by Bhusal et al. who reported no association between time at start of ambulation and development of PSH (18). The difference may be explained by the activities they were using to quantify the start of ambulation such as raising the head, sitting position, and upright posture without considering moving out of bed.

This study found that women who underwent spinal anesthesia via entry at the level of L3-4 were 0.38 times less likely to develop PSH (*p*=0.022) compared to those with entry at L4-5. This is consistent with the findings by Ali Jabbari et al. where the entry level at L4-5 was associated with more PSH as compared to L3-4 [13]. The explanation is the difference in CSF pressure gradient between subarachnoid space and epidural space at the different levels that results in more CSF leakage. This pressure gradient is potentiated even further in an upright position.

This study found that women prescribed weak opioids (*p*=0.002), nonsteroidal anti-inflammatory drugs with nonopioids (*p*=0.025), nonsteroidal anti-inflammatory with weak opioids (*p*=0.044) were 21, 6, and 5 times more likely to develop PSH, respectively, as compared to those with nonsteroidal anti-inflammatory and strong opioids. The explanation may be that as the strength of the analgesics is increased along the analgesics ladder, the less pain one will perceive, camouflaging the appearance of the headache before spontaneous resolution of the PSH occurs.

### 4.1. Study Strength and Limitation

It is the first such study to be undertaken from western Uganda particularly at Fort portal regional referral hospital. The study was a prospective cohort that is suited to study associations. The principal investigator was not technically able to perform the procedure and relied more on own observation and information from the procedurist. This was overcome by strict following of study methodology.

## 5. Conclusions

The incidence of postspinal headache among obstetric mothers undergoing spinal anesthesia at Fort Portal Regional Referral Hospital is high as compared to the global incidence. Postspinal headache is significantly associated with increased risk of PSH if a cutting spinal needle is used, allowing a higher amount of CSF to fall, an increased number of puncture attempts, a prior history of chronic headache, a higher amount of intraoperative blood loss, late postoperative ambulation, and inadequate analgesics. By using a smaller gauge needle, preventing CSF loss, making deliberate attempts to ensure a successful puncture attempt with fewer attempts, reducing intraoperative blood loss, encouraging earlier ambulation, and prescribing adequate analgesia can reduce the incidence and may be the severity of postspinal headache. A high body mass index, puncture at L3-4 level, and adequate analgesia were found to reduce the risk. Maintaining a higher than average BMI and fewer puncture attempts at L3-4 is also protective.

## Figures and Tables

**Table 1:** Factors associated with postspinal headache.

Variables	Categories	^c^OR (95% CI)	*p*	^a^OR (95% CI)	*p*
Ag	<17 years	0.741 (0.28–1.963)	0.540		
	18–30 years	1.096 (0.61–1.969)	0.759		
	>31 years	1			

Body mass index	>30	1.293 (0.746–2.242)	0.571	2.133 (0.631–7.207)	0.223
	25–29.9	0.511 (0.36–0.724)	0.012^*∗*^	0.471 (0.224–0.989)	0.047^*∗*^
	<18.5	2.552 (0.226–28.838)	0.449	0.963 (0.0035–26.149)	0.982
	18.5–24.9	1		1	

Chronic headache	Yes	1.695 (0.900–3.191)	0.102^*∗*^	3.326 (1.409–7.85)	0.006^*∗*^
	No	1		1	

Number of attempts	>3 attempts	8.889 (1.655–47.741)	0.011^*∗*^	20.17 (2.614–155.635)	0.004
	3 attempts	11.457 (4.974–26.387)	<0.001^*∗*^	27.61 (7.671–99.377)	<0.001^*∗*^
	2 attempts	4.148 (2.249–7.650)	<0.001^*∗*^	7.765 (3.48–17.326)	<0.001^*∗*^
	1 attempt	1		1	

CSF allowed to drop	2-3 drops	6.265 (3.064–12.811)	<0.001^*∗*^	3.278 (1.263–8.51)	0.015^*∗*^
	0-1 drop	1		1	

Years of experience anesthetist	<3 years	0.47 (0.204–1.081)	0.075^*∗*^	3.519 (0.991–12.492)	0.052
	3–5 years	0.627 (0.275–1.431)	0.268	3.219 (0.941–11.019)	0.063
	6–10 years	0.759 (0.307–1.876)	0.550	1.461 (0.362–5.901)	0.594
	>10 years	1		1	

Level of entry	L2-3	0.595 (0.327–1.084)	0.090	0.515 (0.225–1.178)	0.116
	L3-4	0.529 (0.293–0.957)	0.035^*∗*^	0.381 (0.167–0.868)	0.022^*∗*^
	L4-5	1		1	

Design of needle	Cutting	1.357 (0.787–2.337)	0.272	3.206 (1.408–7.299)	0.006^*∗*^
	Pencil tip	1		1	

Size of needle	25 G	1.011 (0.514–1.990)	0.974		
	27 G	1			

Type of operation	Emergency	0.864 (0.267–2.797)	0.808		
	Elective	1			

Amount of blood loss	>1500 ml	5.371 (1.735–16.625)	0.004^*∗*^	6.618 (1.582–27.687)	0.010^*∗*^
	>1000−1500 ml	1.058 (0.493–2.270)	0.885	1.728 (0.579–5.155)	0.327
	500−1000 ml	2.25 (1.282–3.948)	0.005^*∗*^	1.858 (0.867–3.986)	0.111
	<500 ml	1		1	

Start of ambulation	>24 hours	1.900 (1.089–3.315)	0.024^*∗*^	2.346 (1.079–5.102)	0.032^*∗*^
	6–12 hours	0.950 (0.085–10.661)	0.967	0.465 (0.023–9.197)	0.615
	12−24 hours	1		1	

Class of analgesics prescribed	Tramadol	6.375 (1.348–30.142)	0.019^*∗*^	20.745 (2.964–145.212)	0.002^*∗*^
	Diclofenac/Panadol	3.945 (1.103–14.115)	0.035^*∗*^	6.104 (1.257–29.651)	0.025^*∗*^
	Diclofenac and tramadol	3.276 (0.900–11.930)	0.072^*∗*^	5.149 (1.047–25.326)	0.044^*∗*^
	>3 classes	5.667 (0.273–117.448)	0.262	12.522 (0.168–930.939)	0.25
	Diclofenac, morphine/pethidine	1		1	

^*∗*^ *p* ≤ 0.05; *p*, level of significance; ^c^OR, crude odds ratio; ^a^OR, adjusted odds ratio; CI, confidence interval.

## Data Availability

The data that support the findings of this study are available from the KIU-WC library, but restrictions apply to the availability of these data, which were used under license for the current study and are not publicly available. Data are, however, available from the authors upon reasonable request and with permission from (Dr. Mohamud Jelle Osman, e-mail: jelle40@yahoo.com).

## Abbreviations

BMI: Body mass index
CSF: Cerebro-spinal fluid
FREC: Faculty Research Ethics Committee
FRRH: Fort Portal Regional Referral Hospital
PDPH: Postdural puncture headache
PSH: Postspinal headache
REC: Research Ethics Committee.

## Additional Points

*Privacy and Confidentiality.* Participants' identification was by codes and initials of their names. Patient assessment was done in a private and secured room. Respondents' information was kept under lock and key throughout the study period and up to 3 months later. Information on the research respondent was not shared with others without their consent and knowledge.
